# Postoperative recurrent patterns of gallbladder cancer: possible implications for adjuvant therapy

**DOI:** 10.1186/s13014-022-02091-6

**Published:** 2022-07-07

**Authors:** Zhijun Yuan, Yongjie Shui, Lihong Liu, Yinglu Guo, Qichun Wei

**Affiliations:** Department of Radiation Oncology, Ministry of Education Key Laboratory of Cancer Prevention and Intervention, The Second Affiliated Hospital, Zhejiang University School of Medicine, Hangzhou, Zhejiang China

**Keywords:** Gallbladder cancer, Adjuvant radiotherapy, Target volume, Patterns of recurrence

## Abstract

**Background:**

Gallbladder cancer (GBC) is an uncommon malignancy with high recurrent rate and poor prognosis. This study investigates the recurrent patterns of postoperative GBC, with the aim to guide the adjuvant treatments, including the radiotherapy.

**Methods:**

Retrospectively analyzed the 109 GBC patients who underwent surgery in our institution from January 2013 to 2018. Clinical follow-up revealed 54 recurrent cases, of which 40 had detailed locations of recurrence. The sites of recurrence were recorded and divided into the tumor bed, corresponding lymphatic drainage area, intrahepatic recurrence, and the other distant metastasis.

**Results:**

The median follow-up time is 34 months (IQR: 11–64). The median disease-free survival (DFS) and overall survival (OS) were 48.8 months and 53.7 months, respectively. Through univariate analysis, risk factors for DFS and OS include tumor markers (CA199 and CEA), hepatic invasion, perineural invasion, lymphovascular invasion, TNM staging and tumor differentiation. Through multivariate analysis, risk factors for DFS include hepatic invasion and TNM staging, and for OS is TNM staging only. Of the 40 cases with specific recurrent sites, 29 patients (29/40, 72.5%) had recurrence in the potential target volume of postoperative radiotherapy (PORT), which include tumor bed and corresponding lymphatic drainage area. The common recurrent lymph node groups included abdominal para-aortic lymph node (No.16, 15/29), hepatoduodenal ligament lymph node (No.12, 8/29), retro-pancreatic head lymph node (No.13, 7/29) and celiac axis lymph node (No.9, 4/29). Twenty cases with recurrences inside the potential PORT target volume were accompanied by distant metastasis. Another 11 cases had distant metastasis alone, so totally 31 cases developed distant metastasis (31/40, 77.5%), including 18 cases with hepatic metastasis.

**Conclusion:**

The recurrence and metastasis rates are high in GBC and adjuvant therapy is needed. Up to 75% of the recurrent cases occurred in the potential target volume of postoperative radiotherapy, suggesting that postoperative radiotherapy has the possible value of improving local-regional control. The potential target volume of radiotherapy should include the tumor bed, No.8, No.9, No.11, No.12, No.13, No.14, No. 16a2, No. 16b1 lymph node groups.

## Introduction

Gallbladder cancer (GBC) is an uncommon type of tumor [1], with the highest incidence in biliary tract tumors [2]. The occurrence of GBC is significantly related to chronic gallstone, and the malignancy incidence is positively correlated with the size of gallstone [3]. Other risk factors include gallbladder polyps (> 1 cm), chronic cholecystitis, porcelain gallbladder, anomalous pancreaticobiliary ductal junction and chronic typhoid infection [4–7]. Patients with Early stage of GBC has no obvious typical clinical symptoms [8], and some cases are incidentally discovered during or after surgery [9]. At the time of diagnosis, most patients are with advanced diseases, only 10–30% of patients are surgically resectable [10]. However surgical resection is still the only way of radical cure for gallbladder cancer [11]. As the postoperative recurrence rate and metastasis rate are high, adjuvant therapeutics should should be emphasized that adjuvant therapy can improve survival in GBC patients [12].

The best adjuvant treatment for GBC remains controversial [13]. Only a few retrospective studies and randomized phase III trials on postoperative adjuvant therapy of GBC can be found through PubMed. Postoperative radiotherapy for GBC was proposed by Bosset et al. for the first time14. Todoroki et al. suggested that surgical resection combined with intraoperative radiation therapy with or without external radiation therapy (with 2- and 3-year survival rate of 20.2% and 10.1%) has a better survival rate than surgical treatment alone for stage IV GBC patients (1- and 2-year survival rate of 11.1% and 0%) (*P* < 0.05) [15]. A retrospective analysis of 3187 GBC patients was conducted by Mojica et al., using the Surveillance, Epidemiology and End Results (SEER) database, patients who received adjuvant radiotherapy had a better median survival (14 months vs. 8 months, *P* < 0.001). Among the numbers of predictors, the only favorable factors for survival were local lymph node metastasis (*P* = 0.0001) and liver invasion (*P* = 0.011) [16]. Similarly, Wang et al. obtained a result that adjuvant radiotherapy was one of the prognostic factors [17]. Yang et al. pointed out that the 5-year survival rate and average survival time of GBC patients in the stage III and IV in adjuvant radiotherapy groups were significantly better than those of the control group (*P* < 0.05), but patients in stage II have no significant difference between two groups [18]. In several postoperative chemoradiotherapy studies of GBC, Jeong et al. suggested that adjuvant radiotherapy might have benefit in local control of GBC [19] and most of the studies revealed that adjuvant chemoradiotherapy can achieve a good long-term survival rate [20, 21], especially it may be useful for the tumor patients undergoing R0 resection without lymph node dissection. However, some studies have shown that adjuvant therapy has no significant effect on improving disease-free survival (DFS) [22]. Due to the lack of phase III randomized controlled trials (RCT) data, the role of postoperative adjuvant radiotherapy remains unclear [23, 24]. According to the National Comprehensive Cancer Network (NCCN) Guidelines, postoperative chemoradiotherapy or chemotherapy is feasible for GBC, especially in patients with lymph node-positive disease [25]. Therefore, the recurrent patterns after GBC surgery may provide valuable information for adjuvant therapeutic options.

In this study, failure patterns of 109 postoperative GBC patients were retrospectively analyzed, with the aim to explore the sites with high risk of local recurrence and metastasis. Such information would be of great value to adjuvant therapy, including the design of radiotherapy target volume and the application of systematic therapy.

## Methods

### Patients

The study was approved by the Institutional Review Board of the Second Affiliated Hospital, Zhejiang University School of Medicine (SAHZU). The medical records from January 2013 to 2018 were retrospectively analyzed, including 109 patients who were followed up for at least 2 months after resection. All the patients achieved histopathology diagnosis after surgery. Patients with previous or contemporaneous tumor history were excluded. The clinical variables collected in the retrospective analysis included gender, age, T stage, N stage, clinical TNM staging, tumor differentiation, recurrence time, tumor biomarkers, history of gallstones, bile duct stones, hepatitis, diabetes, hypertension, schistosomiasis and postoperative adjuvant treatment. Tumor clinical staging was performed according to AJCC staging system, 8th Edition. Follow-up period ended on September 28, 2020.

### Recurrent patterns

According to the 8th edition of the AJCC staging system, inter-aortocaval lymph node and para-aortic lymph node metastasis are regarded as distant metastasis. Therefore, in this study, the term “local-regional recurrence” was not used. We defined “the potential target volume of postoperative radiotherapy (PORT)” which include the tumor bed (surgical margin) and high-risk lymphatic drainage area. Long term follow-up was performed by specialist physicians. The initial recurrent sites were classified to the potential target volume of PORT recurrence, intrahepatic recurrence, and other distant recurrence. Tumor recurrences were detected by imagiological examination (most cases will be monitored by continuous postoperative evaluation). For recurrent local lymph nodes, it can have the features of greater or equal to 10 mm in short diameter, obvious necrosis, obvious enhancement, and eccentric calcification. If possible, PET/CT can assist in diagnosis, and the presence of distant metastasis can be determined at the same time. There are only 2 cases were diagnosed as tumor recurrence by biopsy, though we encouraged to get definite pathological diagnosis. The initial recurrent pattern, DFS and OS will be analyzed. DFS was from the day of operation to the time of first tumor recurrence, OS was from the day of operation to the death or the last follow-up time.

### Statistical analysis

The correlation of patients’ characteristics with DFS and OS were analyzed by Kaplan Meier analysis. Significance was evaluated with the log-rank test. Cox proportional hazards models was applied for multivariate survival analysis. Statistical significance was defined as the *P* value < 0.05. IBM SPSS Statistics 23.0 and Graphpad Prism 5.0 was used for statistical analyses.

## Results

### Patient characteristics

The characteristic statistics of 109 patients are shown in Table 1. There were 37 males (33.9%) and 72 females (66.1%). The mean age was 64.5 years, and the median age was 65 years (IQR: 56–73). Preoperative CA-199 and CEA elevation were found in 48.6% (52/107 cases) and 24.8% (26/105 cases) of the cases. Among the 109 patients, 61 patients (56.0%) had gallstones. As to the pathological differentiation of GBC, 20 cases (18.3%) were well differentiated, 35 cases (32.1%) were moderately differentiated, 45 cases (41.3%) were poorly differentiated, 1 case (0.9%) was undifferentiated, and the differentiation of the other 8 cases (7.3%) were unknown. According to the 8th edition of AJCC Staging System, the tumor T stage was as follows: Tis, 6 cases (5.5%); T1, 10 cases (9.2%); T2, 50 cases (45.9%); T3, 35 cases (32.1%); T4, 8 cases (7.3%). N stage: N0, 67 cases (61.5%); N1, 26 cases (23.9%); N2, 16 cases (14.7%). Distant metastasis occurred in 9 cases (8.3%). In TNM staging, there were 6 (5.5%), 10 (9.2%), 31 (28.4%), 36 (33%) and 26 cases (23.9%) with stage 0, I, II, III and VI respectively. Postoperative pathology showed liver invasion in 30 cases (27.5%), perineural invasion in 33 cases (30.3%), lymphovascular invasion in 36 cases (33%).

**Table 1:** Gallbladder cancer patient characteristics

Characteristics		No	Percentage (%)
Gender	Male	37	33.9
	Female	72	66.1
Age (yrs)	Median	65	
	Range	37–89	
CA199 (107 cases)	> 37 U/ml	52	48.6
CEA (105 cases)	> 5 ng/ml	26	24.8
Gallstone		61	56.0
Bile duct stone		20	18.3
Hepatitis		5	4.6
Hypertension		37	33.9
Diabetes		7	6.4
History of schistosomiasis		5	4.6
Jaundice	Serum bilirubin > 17.1 μmol/L	29	26.6
	Serum bilirubin > 34.2 μmol/L	16	14.7
T stage	Tis	6	5.5
	T1	10	9.2
	T2	50	45.9
	T3	35	32.1
	T4	8	7.3
N stage	N0	67	61.5
	N1	26	23.9
	N2	16	14.7
Distant metastasis		9	8.3
AJCC 8th Staging System	0	6	5.5
	I	10	9.2
	II	31	28.4
	III	36	33.0
	IV	26	23.9
Liver invasion		30	27.5
Perineural invasion		33	30.3
Lymphovascular invasion		36	33.0
Tumor differentiation	Well differentiated	20	18.3
	Moderate differentiated	35	32.1
	Poor differentiated	45	41.3
	Undifferentiated	1	0.9
	Unknown	8	7.3
Adjuvant chemotherapy		8	7.3
Adjuvant chemoradiotherapy		5	4.6

### Follow-up and survival

**Fig. 1:**
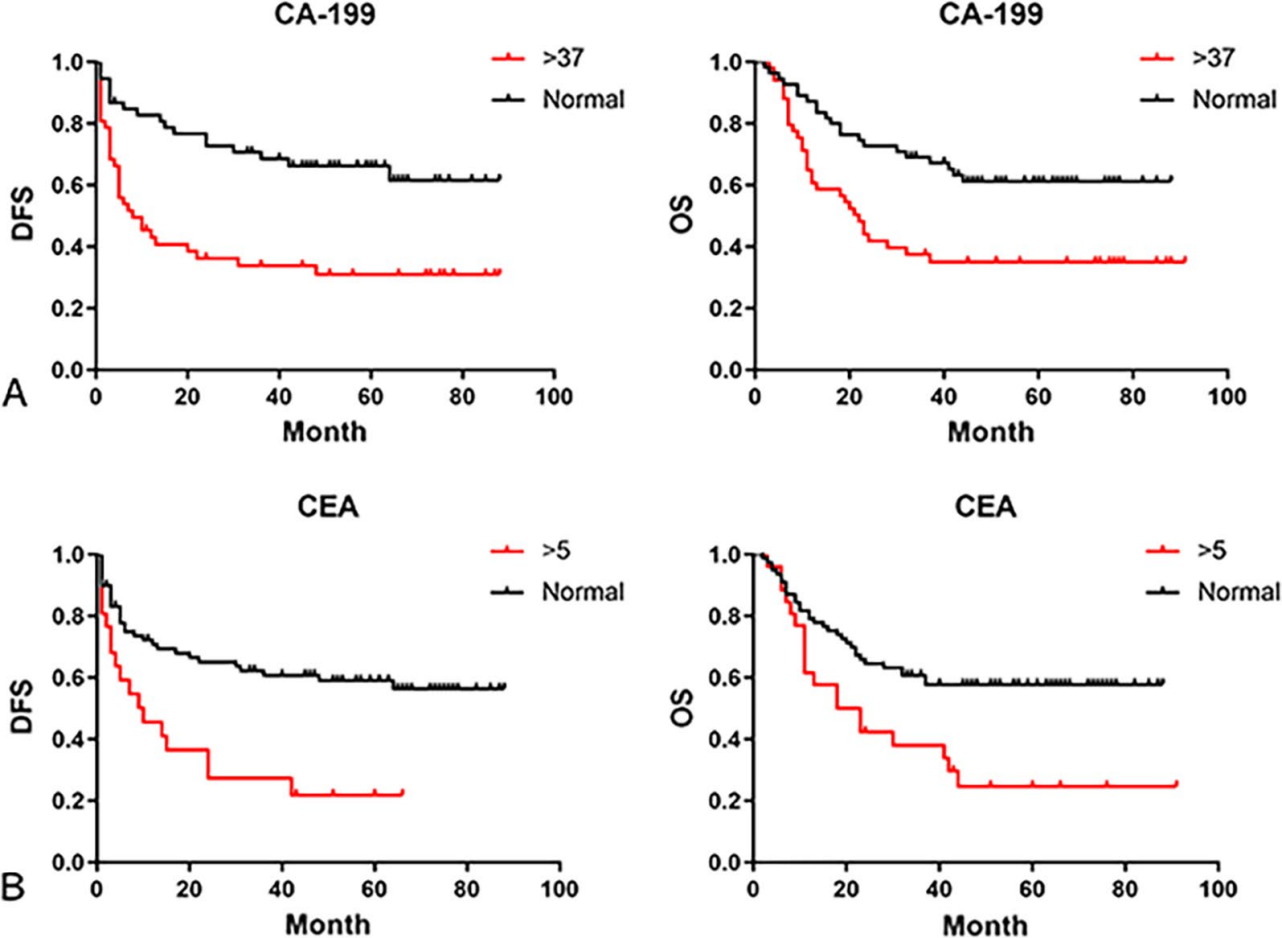
Survival outcomes of baseline data. (**A**) Kaplan–Meier curves of DFS and OS in group with CA-199 elevated or normal. (**B**) Kaplan–Meier curves of DFS and OS in group with CEA elevated or normal

**Fig. 2:**
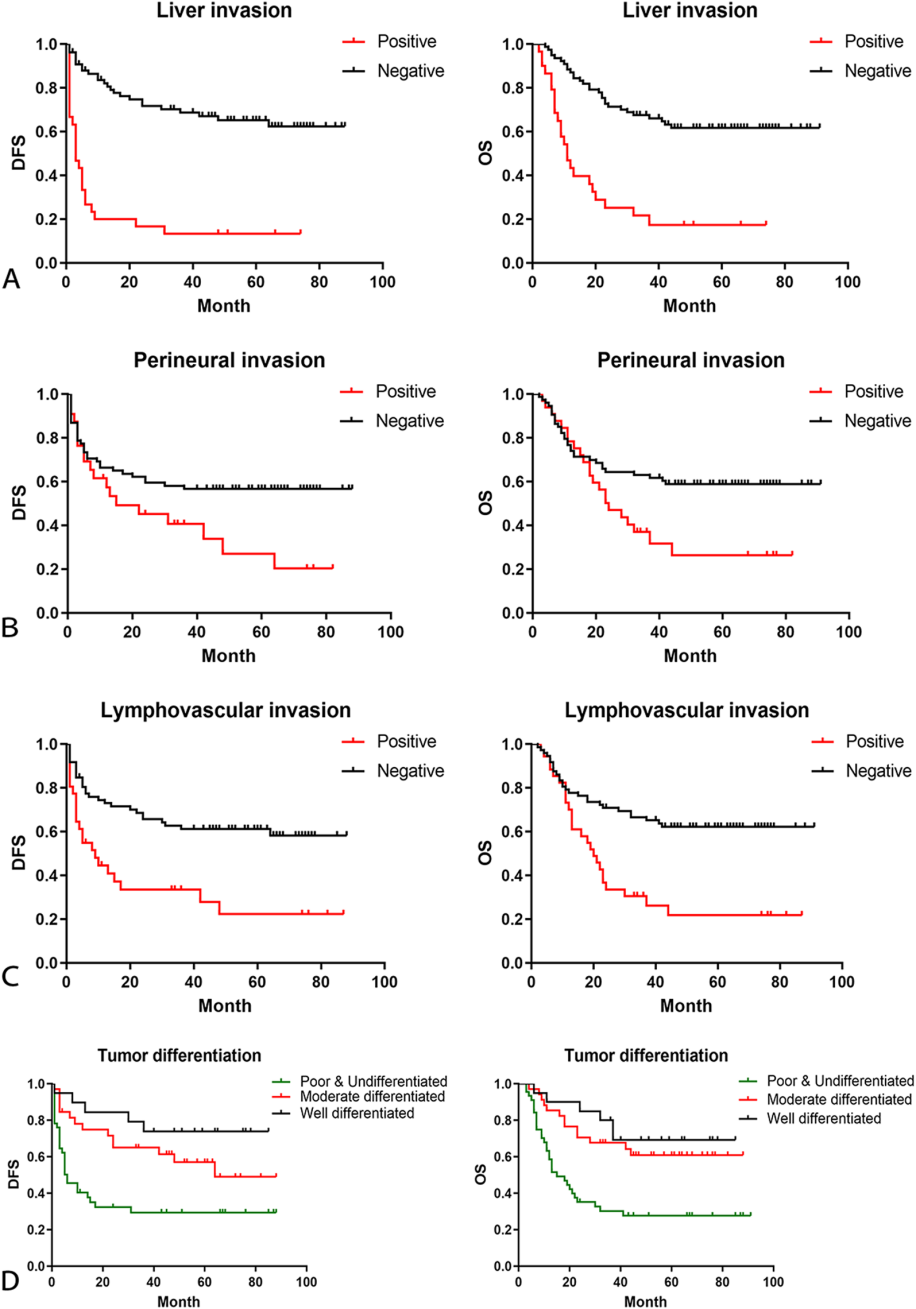
Survival outcomes of pathological factors. (**A**) Kaplan–Meier curves of DFS and OS in group with liver invasion positive or negative. (**B**) Kaplan–Meier curves of DFS and OS in group with perineural invasion positive or negative. (**C**) Kaplan–Meier curves of DFS and OS in group with lymphovascular invasion positive or negative. (**D**) Kaplan–Meier curves of DFS and OS in group of tumor differentiation

**Fig. 3:**
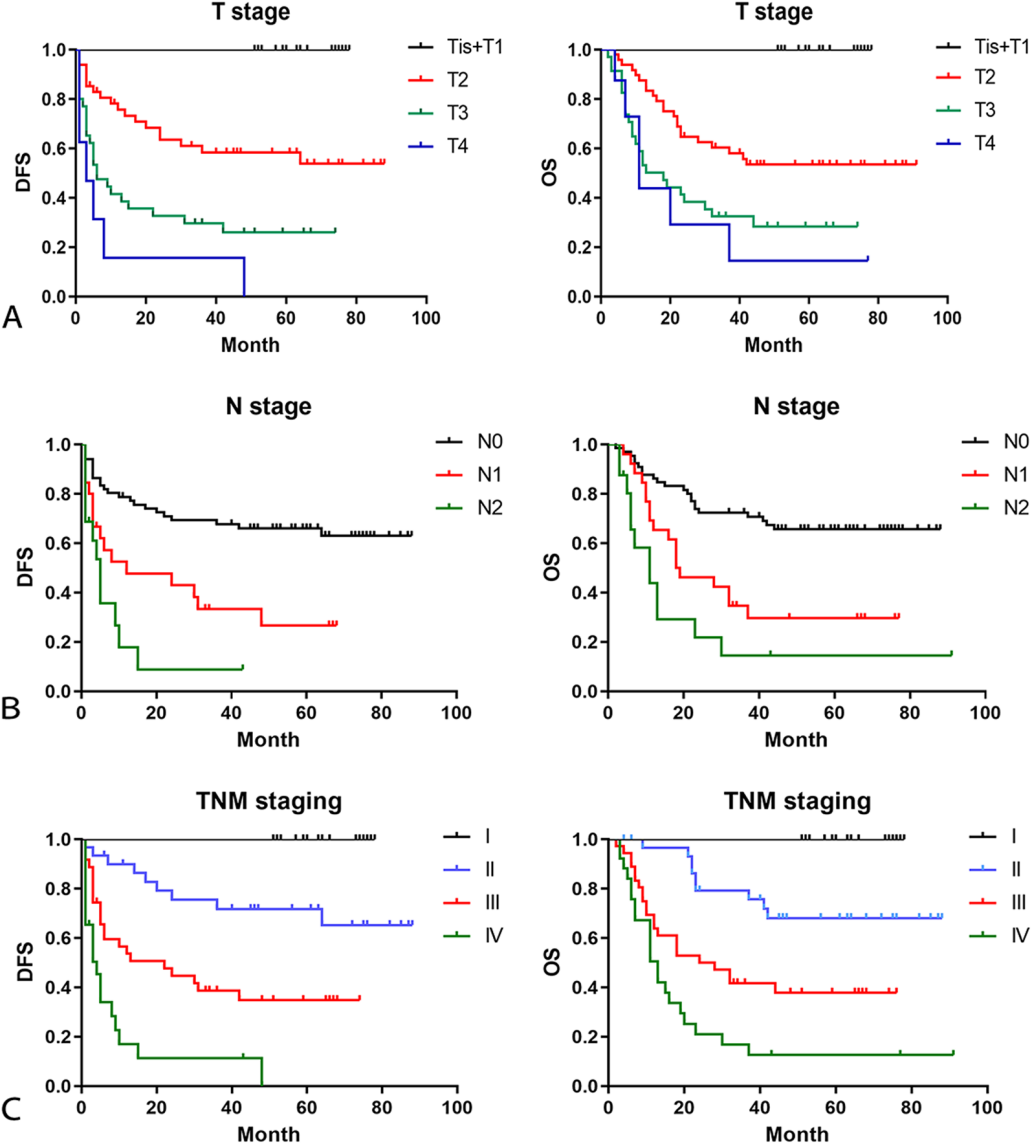
Survival outcomes of tumor staging. (**A**) Kaplan–Meier curves of DFS and OS in group of T stage. (**B**) Kaplan–Meier curves of DFS and OS in group of N stage. (**C**) Kaplan–Meier curves of DFS and OS in group of TNM staging

The median follow-up time was 34 months (IQR: 11–64) in 109 patients. The median disease free survival (DFS) and overall survival (OS) were 48.8 months and 53.7 months. In univariate analysis, there was no significant difference in the prognosis of GBC patients with gender, age, preoperative jaundice and previous medical history. The postoperative survival status of patients was related to the elevation of CA199 (DFS, *P* = 0.0002; OS, *P* = 0.0029) and CEA (DFS, *P* = 0.0009; OS, *P* = 0.0064) (Fig. 1). The other factors included liver invasion (DFS, *P* < 0.0001; OS, *P* < 0.0001), perineural invasion (DFS, *P* = 0.0412; OS, *P* = 0.0169), lymphovascular invasion (DFS, *P* = 0.0004; OS, *P* = 0.0003), tumor differentiation (well vs moderate vs poor and undifferentiated, DFS, *P* = 0.0006; OS, *P* = 0.0002) (Fig. 2), tumor T stage (Tis and T1 vs. T2 vs. T3 vs. T4, DFS, *P* < 0.0001; OS, *P* < 0.0001), lymph node metastasis N stage (N0 vs. N1 vs. N2, DFS, *P* < 0.0001; OS, *P* < 0.0001) and the tumor TNM staging (I vs. II vs. III vs. IV, DFS, *P* < 0.0001; OS, *P* < 0.0001) (Fig. 3). The results of all univariate analysis are shown in Table 2. In multivariate analysis, both liver invasion (RR: 2.308; 95% confidence interval [CI]: 1.045–5.097; *p* = 0.038) and TNM staging of tumor (RR: 3.135; 95% confidence interval [CI]: 1.432–6.863; *p* = 0.004) are important to PFS. But for OS, only TNM staging (RR: 2.676; 95% confidence interval [CI]: 1.396–5.132; *p* = 0.003) is statistically significant.

**Table 2:** Univariate analysis of PFS and OS in gallbladder cancer

Characteristics		No	Percentage (%)	Median DFS	*P* value	Median OS	*P* value
Gender	Male	37	33.9	Not reached	0.5023	Not reached	0.396
	Female	72	66.1	36		41	
Age (yrs)	< 65 years old	53	48.6	Not reached	0.6539	Not reached	0.0707
	≥ 65 years old	56	51.4	24		32	
CA199 (107 cases)	> 37 U/ml	52	48.6	8	**0.0002**	22	**0.0029**
	Normal	55	51.4	Not reached		Not reached	
CEA (105 cases)	> 5 ng/ml	26	24.8	10	**0.0009**	20.5	**0.0064**
	Normal	79	75.2	Not reached		Not reached	
Gallstone	Positive	61	56.0	64	0.6478	37	0.5273
	Negative	48	44.0	39.5		Not reached	
Bile duct stone	Positive	20	18.3	Not reached	0.0778	Not reached	0.3589
	Negative	89	81.7	30		42	
Hepatitis	Positive	5	4.6	5	0.5023	15	0.3835
	Negative	104	95.4	64		Not reached	
Hypertension	Positive	37	33.9	42	0.8149	44	0.6685
	Negative	72	66.1	64		Not reached	
Diabetes	Positive	7	6.4	Not reached	0.7271	Not reached	0.3616
	Negative	102	93.6	48		42	
Schistosomiasis	Positive	5	4.6	Not reached	0.3306	Not reached	0.7756
	Negative	104	95.4	48		44	
Jaundice	Serum bilirubin > 17.1 μmol/L	29	26.6	Not reached	0.5973	32	0.3446
	Normal	80	73.4	48		Not reached	
T stage	Tis + T1	16	14.7	Not reached	** < 0.0001**	Not reached	** < 0.0001**
	T2	50	45.9	Not reached		Not reached	
	T3	35	32.1	6		18	
	T4	8	7.3	3		11	
N stage	N0	67	61.5	Not reached	** < 0.0001**	Not reached	** < 0.0001**
	N1	26	23.9	12		18	
	N2	16	14.7	5		11	
TNM Staging	II	31	28.4	Not reached	** < 0.0001**	Not reached	** < 0.0001**
	III	36	33.0	22		24	
	IV	26	23.9	4		13	
Liver invasion	Positive	30	27.5	3	** < 0.0001**	11	** < 0.0001**
	Negative	79	72.5	Not reached		Not reached	
Perineural invasion	Positive	33	30.3	15	**0.0412**	24	**0.0169**
	Negative	76	69.7	Not reached		Not reached	
Lymphovascular invasion	Positive	36	33.0	9	**0.0004**	20	**0.0003**
	Negative	73	67.0	Not reached		Not reached	
Tumor differentiation	Well	20	18.3	Not reached	**0.0006**	Not reached	**0.0002**
	Moderate	35	32.1	64		Not reached	
	Poor + Undifferentiated	46	42.2	6		15	

The bold figures in the table are the data with *P* < 0.05

### Recurrence rate

Fifty-four patients (54/109, 49.5%) developed postoperative recurrence at the time of last follow-up. No recurrence was found in patients with Tis and T1. The recurrence rates of patients with T2, T3, T4 were 42.0% (21/50 cases), 71.4% (25/35 cases), 100% (8/8 cases), respectively. The recurrence rates of patients with N0, N1, N2 were 35.8% (24/67 cases), 69.2% (18/26 cases), 75% (12/16 cases). As to TNM staging, the recurrence rates of those with staging 0 & I, II, III, and IV were 0% (0/16 cases), 32.3% (10/31 cases), 63.9% (23/36 cases) and 80.8% (21/26 cases). The specific recurrence rates are shown in Table 3.

**Table 3:** Recurrent pattern of gallbladder cancer (40 cases in total)

Staging	No	Tumor bed (10 cases)	Lymphatic drainage area (23 cases)	Remnant liver (18 cases)	Other distant metastases (21 cases)
*T*					
T0 &T1	16	0 (0%)	0 (0%)	0 (0%)	0 (0%)
T2	50	5 (10%)	10 (20%)	3 (6%)	8 (16%)
T3	35	5 (14.3%)	10 (28.6%)	11 (31.4%)	8 (22.9%)
T4	8	0 (0%)	3 (37.5%)	4 (50%)	5 (62.5%)
*N*					
N0	67	5 (7.5%)	9 (13.4%)	10 (14.9%)	9 (13.4%)
N1	26	5 (19.2)	6 (23.1%)	5 (19.2%)	7 (26.9%)
N2	16	0 (0%)	8 (50%)	3 (18.8%)	5 (31.3%)
*TNM*					
0 & I	16	0 (0%)	0 (0%)	0 (0%)	0 (0%)
II	31	2 (6.5%)	3 (9.7%)	3 (9.7%)	3 (9.7%)
III	36	8 (22.2%)	8 (22.2%)	8 (22.2%)	10 (27.8%)
IV	26	0 (0%)	12 (46.2%)	7 (26.9%)	8 (30.8%)

### Initial disease recurrence

Among the 54 cases, the imaging of 40 cases were able to identify the sites of initial recurrence. The other 14 cases had follow-up imaging in local hospital and came to our institution with hardcopy diagnostic report only, so the exact locations of tumor recurrences were unavailable. Only 2 recurrent cases were diagnosed by biopsy, and the remaining cases were diagnosed as tumor recurrence by serial follow-up imaging.

**Fig. 4:**
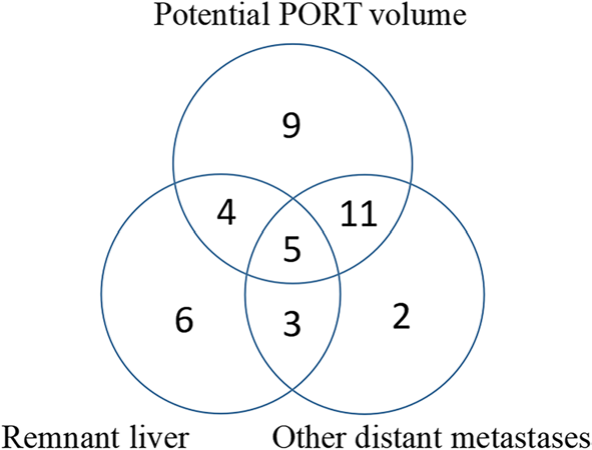
Initial recurrent pattern of GBC. Initial recurrent pattern of 40 cases occurred in the potential PORT volume, remnant liver and the other distant sits

Initial recurrences occurred in the potential PORT volume, remnant liver and the other distant sits were 29 (29/40, 72.5%), 18 (18/40, 45.0%) and 21 (21/40, 52.5%), respectively. Among them, 23 cases had multiple sites of initial disease recurrence (Fig. 4).

Of the 29 patients whose initial recurrence inside the potential PORT volume, 9 (9/40, 22.5%) developed recurrence only inside the potential PORT volume, including 2 tumor bed recurrences, 7 lymph node metastases, and none had recurrence in both sites simultaneously (Table 4). Twenty had synchronous distant metastasis, including 9 in remnant liver, 16 in other distant sites, and of these, 5 had synchronous recurrences at remnant liver and other distant sites.

**Table 4:** The characteristics of 9 patients with isolated PORT volume recurrences

	Postoperative pathological stage	Postoperative pathologically positive lymph nodes	Detailed recurrence sites
Case 1	T3N2M0	12, 13	12 (12a), 7, 16 (16a2)
Case 2	T2N0M0		12 (12b)
Case 3	T2N0M0		Tumor bed
Case 4	T2N0M1 (Hepatic metastasis)		13
Case 5	T2N1M0	9, 13	16 (16b1)
Case 6	T3N2M0	12, 13	3, 6, 13, 17, 7, 8, 9,12, 14, 16 (16a1, 16a2, 16b2)
Case 7	T2N1M1 (Metastatic lymph nodes invade surrounding tissue)	13	16 (16b1, 16b2)
Case 8	T2N1M0	8、9、12	Tumor bed
Case 9	T3N1M1 (Metastatic lymph nodes invade surrounding tissue)	13	13, 15, 16 (16a2, 16b1)

Among the 31 patients with distant metastasis, 11 patients did not develop recurrence in the potential PORT volume, including 6 hepatic metastasis, 1 abdominal wall metastasis, 1 multiple pelvic implantation metastasis and 3 multiple metastasis in remnent liver and abdominal wall. The other 20 patients had distant metastasis and recurrence in the potential PORT volume at the same time.

In the 23 cases of lymph node recurrence, the common sites of lymph node metastases were para aortic lymph nodes (station No.16, n = 15), hepatoduodenal ligament lymph nodes (No.12, n = 8), retro-pancreatic head lymph nodes (No.13, n = 7), celiac axis lymph nodes (No.9, n = 4). In addition, 3 cases had recurrence in the right lymph nodes of cardia (No.1), lesser curvature lymph nodes (No.3), splenic artery lymph nodes (No.11) and superior mesenteric artery and vein lymph nodes (No.14). There were 2 cases in superior pyloric lymph nodes (No.5), left gastric artery lymph nodes (No.7), middle colon vascular lymph nodes (No.15) and anterior pancreatic lymph nodes (No.17). And 1 case occurred in gastric omental lymph nodes (No.4), subpyloric lymph nodes (No.6), common hepatic artery lymph nodes (No.8) and splenic hilum lymph nodes (No.10). In 15 cases of para aortic lymph node recurrence, there were 2 cases in 16a2, 3 cases in 16b1, 7 cases in 16a2 and 16b1, 1 case in 16b1 and 16b2, 1 case in 16a1, 16a2 and 16b2, and 1 case in 16a2, 16b1 and 16b2 at the same time.

## Discussion

The study showed the factors affecting the survival status of patients with gallbladder cancer after resection and the recurrence mode after operation. According to the research results, 49.5% of the patients had recurrence after surgery, which is consistent with the recurrence rate of some previous studies [26, 27].

In this study, postoperative recurrence rate varied with different stages. Postoperative recurrence mainly occurs in the middle and advanced stages of gallbladder cancer. For early gallbladder cancer (TNM stage 0&I), the postoperative recurrence rate is 0%, and the five-year survival rate is 100%. This result was the same as that of lee et al. [28]. Of the 40 cases with detail follow-up records of recurrence, there was no significant difference between relapsing in radiotherapy potential volume only (9 cases, 22.5%) and distant metastasis only (11 cases, 27.5%). According to the different recurrent patterns of previous trials, this result is consistent with the fact [22, 26, 29, 30]. Although there is no plentiful enough large-scale phase III clinical trial, many previous trials and this study suggested that specific adjuvant therapy should be taken for GBC with high recurrence rate.

Postoperative radiotherapy is recommended to combine with chemotherapy in all feasible patients with locally advanced gallbladder cancer. According to the analysis of the initiate recurrent pattern of 29 cases, the common recurrent lymph nodes were para aortic lymph nodes (No.16), hepatoduodenal ligament lymph nodes (No.12), retro-pancreatic head lymph node (No.13), celiac axis lymph node (No.9), right lymph node of cardia (No.1), lesser curvature lymph node (No.3), splenic artery lymph node (No.11) and superior mesenteric artery and vein lymph node (No.14). According to Uesaka and Ito's anatomical description of gallbladder lymphatic drainage, there were four routes of gallbladder lymphatic drainage: gallbladder pancreaticoduodenal route (mainly), gallbladder hepatoduodenal ligament route, gallbladder mesenteric route and porta hepatis route [31, 32]. Finally, the first three pathways converged at the level of the left renal vein in the para aortic lymph nodes. The porta hepatis pathway may be related to liver metastasis. The left renal vein is the dividing line between No.16a2 and No.16b1, which is consistent with the recurrent pattern of para aortic lymph nodes in 15 cases. Lymph nodes recurred at the level of left renal vein in every case. In addition, a small number of No.16a1 and No.16b2 recurrences were considered as secondary lymph node metastasis.

J Socha et al. performed systematic review and meta-analysis of lymph node metastases in biliary tumors of different T stages [33]. It is suggested that the adjuvant radiotherapy target volume of T3-4 GBC should include No.8, No.9, No.12, No.13, No.14, No.16 lymph node groups. In our retrospective study, only 1 case of recurrent metastatic lymph node was located at No.8. For cross-regional lymph nodes, the center of lymph node was used as the localization marker for statistical analysis during imaging review. The No.9, No.12 and No.13 were all distributed around No.8, and each landmark tissues and organs were close to each other. Therefore, combined with the literature, No.8 should be included in the postoperative radiotherapy target volume. Combined with other research reports and structural relationships, No.1 and No.3 have a small probability of metastasis, and the greater toxic side effects of PORT can be predicted. So the inclusion of the above two groups of lymph nodes in the PORT target volume is not considered in this study. No.14 metastases of locally advanced gallbladder cancer have also been observed in other studies, and the same number of No.11 metastases occurred in our study. Anatomically, we considered that the PORT target volume should include No.11 (corresponds to the lymphatic region of the proximal splenic artery) and No.14, which may improve local control of the disease. At the same time, attention should be paid to patients' radiotherapy tolerance, and appropriate adjustments can be made for different patients and different stages. Therefore, after comprehensive consideration, the target volume of adjuvant radiotherapy should include the tumor bed, No.8, No.9, No.11, No.12, No.13, No.14, No.16a2 and No.16b1 lymph node groups.

Among the GBC patients with TNM stage II-IV (93 cases, 54 cases of recurrence, recurrence rate: 58%), the recurrence rate in the potential target volume of radiotherapy is almost the same as the rate of distant metastasis. Therefore, adjuvant chemotherapy is also important. In this study, OS and DFS of adjuvant radiotherapy, adjuvant chemotherapy and adjuvant chemoradiotherapy were not statistically significant. The same results were also found in other trials [34, 35]. This result may be caused by the small sample size. In two phase III clinical trials, capecitabine monotherapy and adjuvant chemotherapy with fluorouracil and mitomycin C, respectively, showed significant improvements in PFS and OS [35, 36]. For the high recurrence rate of GBC, as well as the high incidence of distant metastasis and local recurrence, postoperative adjuvant chemoradiotherapy is a better choice, especially for the relatively advanced GBC. The phase II SWOG S0809 trial included patients with extrahepatic cholangiocarcinoma or gallbladder cancer (N = 79), providing prospective data for adjuvant chemotherapy/chemoradiotherapy (concurrent chemoradiotherapy with capecitabine after capecitabine/gemcitabine chemotherapy). The 2-year survival rate was 65%, and the median survival time was 35 months. Most of the patients (86%) who participated in the trial completed the treatment, indicating that the regimen was generally tolerable [37, 38]. But gemcitabine concurrent chemoradiotherapy is not recommended because of its limited experience and side effects [39].

According to the National Comprehensive Cancer Network (NCCN) Guidelines, in addition to gemcitabine + cisplatin and other chemotherapy regimens, targeted therapy or immunotherapy can be combined for postoperative recurrence of gallbladder cancer in special circumstances. For NTRK gene fusion-positive tumors, Entrectinib, Larotrectinib can be used. For MSI-H/dMMR tumors, Pembrolizumab can be used.

## Conclusion

This study analyzed failure patterns after surgery for gallbladder cancer. About half of the patients will relapse after surgery, of which about 75% recurrences occur in the potential volume of radiotherapy, and there are almost the same number of distant recurrences. Therefore, adjuvant chemoradiotherapy is necessary for patients with advanced gallbladder cancer. For adjuvant radiotherapy, the target volume should include the tumor bed, No.8, No.9, No.11, No.12, No.13, No.14, No.16a2, No.16b1 lymph node groups. For relatively rare cancer species, adjuvant radiotherapy for gallbladder cancer may need multicenter prospective trials to prove its effectiveness.

## Data Availability

Datasets can be retrieved from authors by formal request from interested readers. Datasets will not be directly shared on public link as the national personal data protection act.

## Abbreviations

GBC: Gallbladder cancer
PET/CT: Positron emission tomography/computed tomography
DFS: Disease-free survival
OS: Overall survival
PORT: Postoperative radiotherapy
SEER: Surveillance, epidemiology and end results
NTRK: Neurotrophic tryrosine receptor kinase
MSI-H: Microsatellite instability-high
dMMR: Deficient mismatch repair
